# Versatile optical manipulation of trions, dark excitons and biexcitons through contrasting exciton-photon coupling

**DOI:** 10.1038/s41377-023-01338-5

**Published:** 2023-12-07

**Authors:** Zhe Li, Xin-Yuan Zhang, Rundong Ma, Tong Fu, Yan Zeng, Chong Hu, Yufeng Cheng, Cheng Wang, Yun Wang, Yuhua Feng, Takashi Taniguchi, Kenji Watanabe, Ti Wang, Xiaoze Liu, Hongxing Xu

**Affiliations:** 1 School of Physics and Technology, Center for Nanoscience and Nanotechnology, and Key Laboratory of Artificial Micro- and Nanostructures of Ministry of Education, Wuhan University, 430072 Wuhan, China; 2 Wuhan University Shenzhen Research Institute, 518057 Shenzhen, China; 3 Institute of Advanced Synthesis, School of Chemistry and Molecular Engineering, Nanjing Tech University, 211816 Nanjing, China; 4 International Center for Materials Nanoarchitectonics, National Institute for Materials Science, 1-1 Namiki, 305-0044 Tsukuba, Japan; 5 Research Center for Functional Materials, National Institute for Materials Science, 1-1 Namiki, 305-0044 Tsukuba, Japan; 6 Wuhan Institute of Quantum Technology, 430206 Wuhan, China; 7 School of Microelectronics, Wuhan University, 430072 Wuhan, China; 8 Henan Academy of Sciences, 450046 Zhengzhou, China

**Keywords:** Optical physics, Optical materials and structures, Optical techniques

## Abstract

Various exciton species in transition metal dichalcogenides (TMDs), such as neutral excitons, trions (charged excitons), dark excitons, and biexcitons, have been individually discovered with distinct light-matter interactions. In terms of valley-spin locked band structures and electron-hole configurations, these exciton species demonstrate flexible control of emission light with degrees of freedom (DOFs) such as intensity, polarization, frequency, and dynamics. However, it remains elusive to fully manipulate different exciton species on demand for practical photonic applications. Here, we investigate the contrasting light-matter interactions to control multiple DOFs of emission light in a hybrid monolayer WSe_2_-Ag nanowire (NW) structure by taking advantage of various exciton species. These excitons, including trions, dark excitons, and biexcitons, are found to couple independently with propagating surface plasmon polaritons (SPPs) of Ag NW in quite different ways, thanks to the orientations of transition dipoles. Consistent with the simulations, the dark excitons and dark trions show extremely high coupling efficiency with SPPs, while the trions demonstrate directional chiral-coupling features. This study presents a crucial step towards the ultimate goal of exploiting the comprehensive spectrum of TMD excitons for optical information processing and quantum optics.

## Abstract (web summary)

This work demonstrates versatile light manipulation of various excitons in WSe_2_ via contrasting exciton-photon coupling with dipole-orientation, diffusion and chirality dependences.

## Graphical abstract

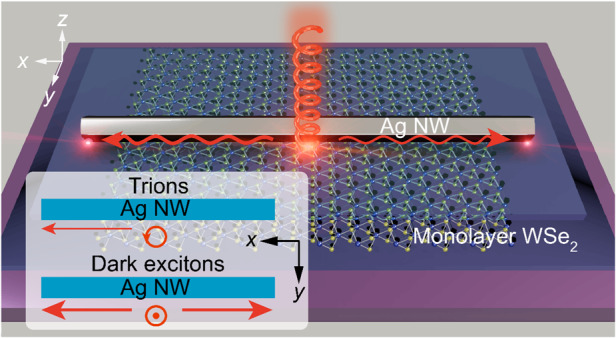

## Introduction

In the monolayer limit, the strong Coulomb interactions and direct band gaps in transition metal dichalcogenides (TMDs) result in tightly bound excitons with striking optical signatures^1–6^. Excitons in the monolayers possess large binding energies of a few-hundred meV and are stable at room temperature. More interestingly, emerging exciton species, such as trions and biexcitons, are formed and spectrally separated with bound multiple-particle configurations^7–13^, offering many opportunities to investigate many-body interactions and related quantum phenomena. Meanwhile, some unique excitonic effects have been discovered because of spin-valley locking band structures in TMD monolayers^14–20^. For the conservation of spin angular momentum, optical transitions of direct-gap excitons in K valleys can occur only with specific circular polarizations^17,21^. This polarization selection rule is also referred to valley polarization, acting as the core mechanism for the booming research of valleytronics. On the other hand, optical transitions of some excitons are spin-forbidden, leading to the discovery of dark excitons in tungsten-based TMD monolayers. The dark excitons are found to hold a much longer lifetime of a few nanoseconds than spin-allowed bright excitons, and unexpectedly their transition dipoles are oriented along the out-of-plane direction^22^. In TMD monolayers, these exciton species of spectral separations, different transition dipoles, unique valley polarization dependence, and distinct carrier dynamics provide flexible approaches to control the emission light with degrees of freedom (DOFs) such as frequency, intensity, polarization, and dynamics.

TMDs have been demonstrated as a versatile platform to manipulate the excitonic emissions with different DOFs when coupled with various photonic nanostructures. In optical cavities, the bright excitonic emission intensities could be significantly amplified with accelerated dynamics by the Purcell effect and even get into the stimulated regime for coherent lasing actions^23–26^. Remarkably, these bright excitons could also reach the strong coupling regime by controlling the coupling strength, giving rise to intriguing polaritonic phenomena^27–35^. In deliberate photonic structures, the excitonic valley polarization could be well preserved and even enhanced^16,30,36,37^; via chiral photonic designs, the valley polarization could be utilized as a novel DOF for sorting and routing optical signals^38–41^. With considerable spectral separations, it is worth noting that different excitons (e.g., trions, dark excitons, and biexcitons) of specific electron-hole configurations provide great opportunities to investigate many-body interactions and related quantum phenomena^9,10,12,42^. Moreover, dark excitons with spin-forbidden transitions are found to couple with photons in a totally different way because of their out-of-plane transition dipoles^22^. The above-mentioned progresses indicate the tremendous potential to exploit TMD excitons with different DOFs, establishing one ultimate goal to exploit the comprehensive spectrum of TMD excitons for optical information processing and quantum optics^43,44^. However, towards this goal, there is still a considerable gap because it remains elusive to fully manipulate all the excitons simultaneously on demand.

In this work, we showcase the optical versatile manipulation of various excitons in a hybrid monolayer WSe_2_-Ag nanowire (NW) structure by harnessing the contrasting photon-exciton interactions with dependences of dipole orientations, diffusion, and chirality. Here the Ag NW is taken as the photonic structure for two-fold reasons: (i) The surface plasmon polaritons (SPPs) in Ag NWs can largely enhance the light-matter interactions for all the exciton species of WSe_2_. The enhancement can be ensured by the highly confined electromagnetic fields; excitons with different transition dipole orientations can all couple to different SPP modes^22,24,31,41,45–47^. (ii) The valley polarization-dependent coupling of excitonic emissions is possible in Ag NWs. By breaking the mode symmetry, the chiral coupling and routing of SPP modes provide a convenient way to manipulate the valley polarized emissions^39^. In this experimental configuration, the excitons (including trions), dark excitons (including dark trions), and biexcitons (including charged biexcitons), are found to couple independently with propagating SPPs in quite different ways. Consistent with the simulations, the dark excitons and dark trions show extremely high coupling efficiency with SPPs, while the trions demonstrate highly directional chiral-coupling features as the valley polarization is present. The detailed experiments and result discussions are elaborated as follows.

## Results

### Design and characterization of the hybrid structure

**Fig. 1:**
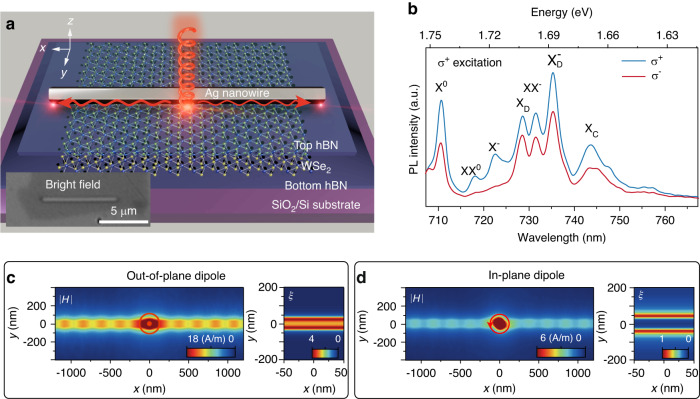
Contrast exciton-photon coupling of various excitons in WSe2. **a** Schematic of the Ag NW-WSe_2_ sample structure. The inset shows the bright field microscopic image. **b** Helicity-resolved PL spectra of monolayer WSe_2_ far away from Ag NW at 4 K under *σ*^+^ excitation at 685 nm. **c** Simulated electromagnetic field (left) and normalized SPP power *ξ* (right) of out-of-plane dipoles coupled with NWs as a function of position, respectively. **d** Simulated electromagnetic field (left) and normalized SPP power *ξ* (right) of in-plane dipoles coupled with NWs as a function of position, respectively

The schematic of the sample structure is shown in Fig. 1a. It consists of an Ag NW with ~8 μm length and a monolayer WSe_2_ encapsulated between two hexagonal boron nitride (hBN) thin films, which are sitting on a SiO_2_/Si substrate (see Methods and Fig. S1 for more details). The exciton species of monolayer WSe_2_ could be distinguished via the hBN encapsulation, as the photoluminescence (PL) spectrum shows in Fig. 1b (pumped by a continuous-wave (CW) laser of 685 nm at 4 K). There appear multiple narrow PL peaks, which are labeled as the neutral A exciton (X^0^), biexciton (XX^0^), trion (X^-^), dark exciton (X_D_), charged biexciton (XX^-^), dark trion $\left({{\rm{X}}}_{{\rm{D}}}^{-}\right)$. The lower energy peaks besides ${{\rm{X}}}_{{\rm{D}}}^{-}$ are considered as exciton complexes (X_C_), which are not the focus of this research^48^. These exciton species are identified by their peak energies, pump-power dependence, and valley polarization (see more details in Figs. S2 and S3), which are consistent with previous reports^42,49–51^. The valley polarization of these excitons is examined with circular polarization degree as *ρ*=(*I*^+^-*I*^-^)/(*I*^+^+*I*^-^), where *I*^+^ and *I*^-^ represent the right and left circular polarized PL intensity. Under right-circular polarized (*σ*^+^) pump light of 685 nm CW laser, the *ρ* of X^0^, XX^0^, and X^-^ are 27%, 79 and 92%, respectively (see Fig. S2 and Table S2 for detailed analysis for the *ρ* of X_D_, XX^-^ and ${{\rm{X}}}_{{\rm{D}}}^{-}$, see Fig. S4 for the *σ*^-^ pump case). Moreover, the sample structure also enables efficient coupling between the SPPs of NWs and the WSe_2_ monolayer. The thin hBN could not only prevent the charge transfer between WSe_2_ and Ag NWs without suppressing the PL quantum efficiency, but also ensure sufficient coupling strength as shown in Fig. 1c, d and Supplementary Fig. S5. The effect of hBN is further verified by the control sample without hBN between the WSe_2_ and Ag NW (Supplementary Fig. S6).

For the distinct properties of these exciton species, their coupling with SPPs of NW occurs in quite different ways. The most distinct properties of these excitons lie at the orientations of transition dipoles. For instance, the dipoles of exciton (X^0^) and trion (X^-^) are in-plane oriented^52^, while those of dark excitons (X_D_) are out-of-plane oriented^22,24,52^. Based on these differences, numerical simulations are carried out to look into their coupling with NW SPPs. In Fig. 1c, the simulated electromagnetic field of an out-of-plane dipole source with NWs is profiled as a function of position (the cross-section electric field distribution is shown in Fig. S7). Along the NWs (*x*-axis) at both directions, the uniform wave-like spread-out indicates the efficient coupling and thus can support long-range propagation towards both ends of the NW. To better characterize the coupling, normalized SPP power *ξ=P/P*_max_ is defined to represent the strength. Along the *y*-axis, however, the coupling becomes more efficient at the NW edges and slightly weaker in the center. When the dipole source goes outside the NWs range, the coupling diminishes drastically. On the contrary, the simulated field of the in-plane dipole source is profiled in Fig. 1d. The field is much weaker than that of the out-of-plane dipole. For the normalized SPP power, the coupling is more efficient around the edge of the NWs and becomes extremely weak at the center.

### Experimental observation of the contrasting exciton-photon coupling

**Fig. 2:**
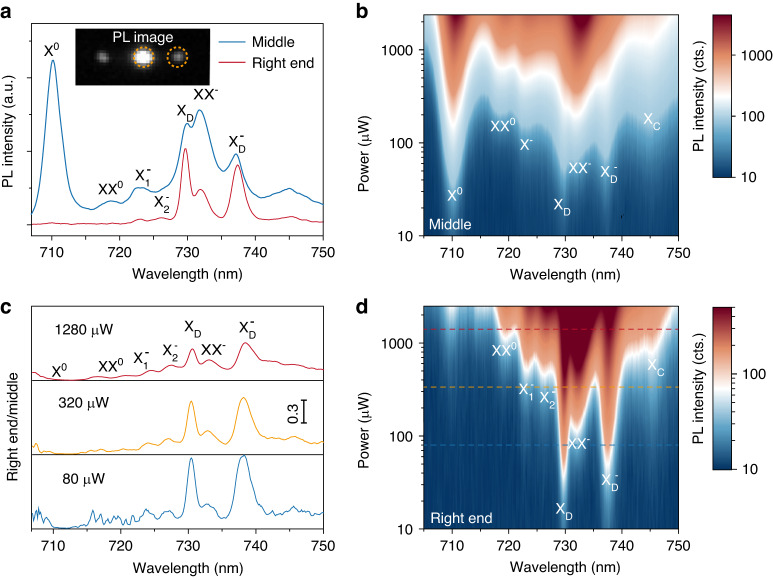
Power-dependent PL spectra from the middle and the right end of the Ag NW. **a** In-situ (collected at the middle point of NW) and propagated PL spectra (collected at right end) with the excitation power of 160 μW (pumped at the middle), the inset shows the corresponding PL image. Color map of in-situ (**b**) and propagated PL spectra (**d**) as a function of pump powers. For better demonstration, the data is acquired at 4 K with *σ*^+^ excitation and *σ*^+^ detection. **c** Spectral coupling efficiency $\kappa$ at the pump powers of 80, 320, and 1280 μW

PL spectroscopy is carried out to observe the coupling features of these different excitons. The pump CW laser of 685 nm is focused onto the middle point of the NW and the PL are collected in the pump area (at the middle) and at both ends of the NW. For convenience, the polarization of pump and PL collections are all set to be right circularly polarized (the polarization analysis is discussed in Figs. 4 and S2). In the PL image of Fig. 2a, the white spots in the middle and at both ends correspond to the scattered signals from the in-situ excitons, and the propagating SPP coupled excitons, respectively. The PL spectra of these two areas are then plotted in Fig. 2a. Apparently, the PL spectrum in the middle is consistent with Fig. 1b, where the exciton (X^0^) emission dominates. In contrast, the PL at the right end shows a totally different profile, where the emissions of dark excitons (X_D_) and dark trions $\left({{\rm{X}}}_{{\rm{D}}}^{-}\right)$ dominate but those of X^0^, X^-^ and XX^0^ decrease drastically. This could be well explained by the simulation of Fig. 1c, where the transition dipoles of X_D_ and ${{\rm{X}}}_{{\rm{D}}}^{-}$ are out-of-plane oriented and are expected to couple more efficiently than those in-plane oriented dipoles of X^0^ and X^-^.

The pump-power dependent PL spectroscopy is taken to further look into the contrasting coupling behaviors. The power-dependent PL spectra at both areas are plotted in Fig. 2b, d (those for the left end are shown in Fig. S8). In the PL spectra of right end, the intensities of X_D_ and ${{\rm{X}}}_{{\rm{D}}}^{-}$ are already quite prominent at low pump powers, and their linewidths are considerably narrower than PL spectra in the middle. As the power increases, other exciton peaks, such as biexcitons (XX^0^) and charged biexcitons (XX^-^), become visible for different power-law dependence. By normalizing the end spectrum (PL intensity *I*_R(L)_ at the right (left) end) with respect to the middle one (PL *I*_M_ in the middle), the coupling efficiency for each exciton species is defined as (*κ*=*I*_R_/*I*_M_). This efficiency $\kappa$ at three typical powers is then summarized in Fig. 2c. It is apparent that the coupling efficiency $\kappa$ is dominant for X_D_ and ${{\rm{X}}}_{{\rm{D}}}^{-}$, which is far larger than all the other excitons which have strong in-plane dipoles. Although the efficiency $\kappa$ decreases as the pump power increases, it is always the unambiguously highest for the X_D_ and ${{\rm{X}}}_{{\rm{D}}}^{-}$. The dominant efficiency $\kappa$ of X_D_ and ${{\rm{X}}}_{{\rm{D}}}^{-}$ is about 2.8 times larger than the $\kappa$ for in-plane dipoles, i.e., the trions under 320 μW pump power. The 2.8 times stronger coupling efficiency is consistent with the simulation, where the coupling strength is calculated to be 2.5 times stronger. Moreover, time-resolved photoluminescence (TRPL) and numerical simulation are carried out to further characterize the efficient coupling between the X_D_, ${{\rm{X}}}_{{\rm{D}}}^{-}$ and Ag NW (Fig. S9). Interestingly, the efficiency $\kappa$ for trions (X^-^) reveals more fine features of the trions. This directly helps resolve two peaks at the original X^-^ position as inter-valley trion $\left({{\rm{X}}}_{1}^{-}\right)$ and intra-valley trion $\left({{\rm{X}}}_{2}^{-}\right)$ because of different valley-indexed three-particle configurations, as reported by the magneto-optical measurements^53,54^. The efficiency $\kappa$ is close to zero for neutral excitons and biexcitons (X^0^ and XX^0^). The case of XX^0^ may be just resulted from the low emission yield and low coupling strength here; the case of X^0^ is attributed to the low coupling strength and much higher propagation loss of SPPs with large re-absorption at the exciton resonance. Due to the narrow absorption linewidth of hBN encapsulated WSe_2_, this SPP loss of re-absorption mainly affects the X^0^ resonance. The light-matter coupling with SPPs thus shows contrasting behaviors for each specific excitons, rendering versatile approaches to control their light emissions.

### Manipulate the light emissions of different excitons by tuning the coupling

Once the light-matter coupling is established for these distinct excitons, their light emissions can be flexibly manipulated by taking advantage of their distinct characteristics. For demonstration, the excitonic emission intensity, and directional coupling with polarization dependence would be deliberately controlled with specific spectral signatures. This controllability is exemplified by exploiting the spectral separations, different excitons’ diffusion lengths and valley DOF.

**Fig. 3:**
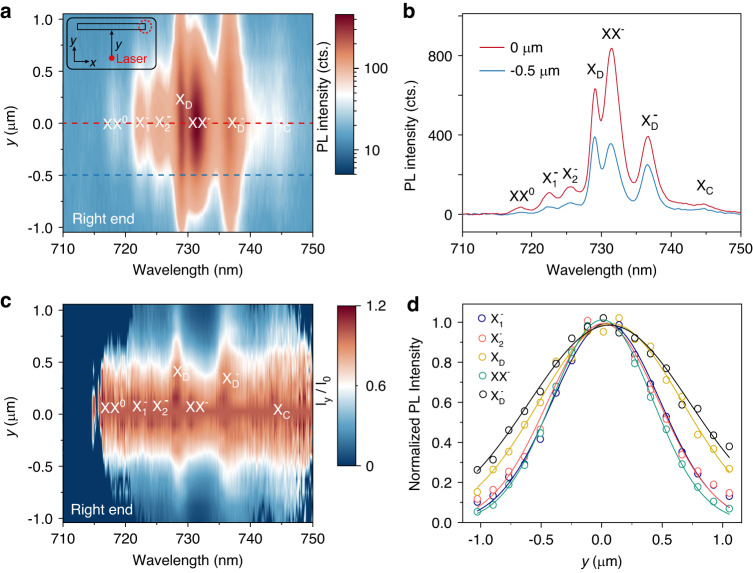
Controlling the light emissions by tuning the exciton-photon coupling. **a** Color map of PL spectra as a function of the pump position *y* under *σ*^+^ excitation and *σ*^+^ detection. The excitation laser is at 685 nm with power of 1 mW. The position *y* corresponds to the coordinates of the axis perpendicular to the axis of NW, and *y* = 0 represents the center point of NW. The inset indicates the scan direction in the sample schematics. **b** PL spectra for *y* = 0 and *y* = −0.5 μm as the line-cuts of red and blue dashed lines in (**a**). **c** Color map of coupling efficiency, *I*_*y*_/*I*_*0*_ as a function of wavelength and position. **d** The normalized PL of 5 typical excitons as a function of *y* position. The dark excitons and dark trions have the largest expansions along the *y* axis

We first demonstrate that the spectral profiles for each exciton can be intentionally altered and even some excitonic emissions can be selectively turned off, as we tune the photonic coupling of different excitons. This is realized by moving the pump spot around the vicinity of the middle point NW. In Fig. 3a, the PL spectra collected at right end (see Fig. S10 for the left end) are mapped by indexing the pump positions along the *y*-axis (the direction perpendicular to the NW axis). As the pump spot moves away from the center point (*y* = 0), X_D_ and ${{\rm{X}}}_{{\rm{D}}}^{-}$ would preserve their emission intensity even at the position *y* > 1.0 μm, while the trions (${{\rm{X}}}_{1}^{-}$ and ${{\rm{X}}}_{2}^{-}$) and charged biexcitons (XX^-^) decrease their intensity much more rapidly. Detailed spectra are shown as the two line-cuts of the PL map at the positions of *y* = 0 and *y* = −0.5 μm as in Fig. 3b. As a function of pump position, the emissions of XX^-^, ${{\rm{X}}}_{1}^{-}$ and ${{\rm{X}}}_{2}^{-}$ can be selectively turned off. To quantitatively analyze this dependence for accurate control, we map out the wide-spread coupling efficiency by normalizing all the spectra with respect to the spectrum at *y* = 0 as in Fig. 3c. With this normalization, X_D_ and ${{\rm{X}}}_{{\rm{D}}}^{-}$ have the most wide-spread efficiency while the XX^-^ has the least. This efficiency is largely determined by the diffusion length, as well as the intrinsic coupling behaviors of the dipole orientations as discussed in Fig. 2. The normalized PL intensity for typical excitons (${{\rm{X}}}_{1}^{-}$, ${{\rm{X}}}_{2}^{-}$, X_D_, ${{\rm{X}}}_{{\rm{D}}}^{-}$ and XX^-^) is then plotted as a function of pump position for diffusion length analysis in Fig. 3d, where a diffusion model is employed to fit the data. As excited by a CW laser, the exciton concentration *n* can be depicted by a simple steady-state diffusion equation^55,56^

1 $$\frac{P{\rm{\alpha }}}{{2{\rm{\pi }}{hvw}}^{2}}{e}^{{-r}^{2}/{w}^{2}}=\frac{n(r)}{{\tau }_{{\rm{X}}}}-{D}_{{\rm{X}}}{{\rm{\nabla }}}^{2}n(r)$$

Where $P$ is the excitation power,$\alpha$ the absorption coefficient at the photo energy *hv*, ${e}^{{-r}^{2}/{w}^{2}}$ the Gaussian profile, *τ*_X_ and *D*_X_ are the exciton lifetime and diffusion coefficient, respectively. The analytical solution to Eq. (1) in a 2D crystal is

2 $$n(r)\propto {\int }_{\!-{{\infty }}}^{{{\infty }}}{K}_{0}({r}^{{\prime} }/{L}_{{\rm{X}}}){e}^{{-\left({r-r}^{{\prime} }\right)}^{2}/{w}^{2}}{dr}^{\prime}$$

where *K*_0_ is the modified Bessel function of the second kind, ${L}_{{\rm{X}}}=\sqrt{{D}_{{\rm{X}}}{\tau }_{{\rm{X}}}}$ is the diffusion length. Equation (2) is employed to obtain the diffusion length in Fig. 3d. The diffusion lengths of X_D_ and ${{\rm{X}}}_{{\rm{D}}}^{-}$ are then estimated to be 0.59 ± 0.10 μm and 0.84 ± 0.13 μm, while those of trions and XX^-^ cannot be quantified as they are too small compared to the beam spot size (see Supplementary S11 for more details). The diffusion length thus provides an efficient approach to tune the exciton-photon coupling to manipulate the spectral profiles of each exciton. Note here that only this SPP coupling with diffusion model can well explain the observed features. The possibility of the in-plane propagation via substrate waveguide is excluded with a detailed discussion in Supplementary Section 12.

**Fig. 4:**
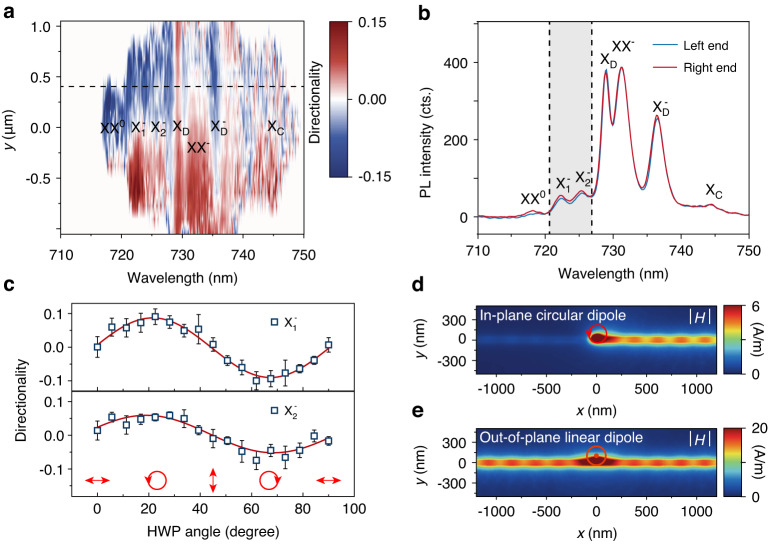
Directional coupling with polarization dependence. **a** Position and wavelength dependent directionality *D* under *σ*^+^ excitation of 685 nm CW laser with 1 mW power. **b** PL spectra from the left and right ends of the Ag NW with *y* ~ 0.4 μm, as indicated by the black dashed line in (**a**). **c** Polarization dependent directionality of ${{\rm{X}}}_{1}^{-}$ and ${{\rm{X}}}_{2}^{-}$, the polarization of the laser is modified by a half wave-plate (HWP). With HWP angle increases, the polarization is set from linear to right/left circular polarization. **d**, **e** are magnetic field distributions of SPPs excited by an in-plane circular dipole source and out-of-plane linear dipole source at *y* = 30 nm, respectively

To demonstrate the control of polarization DOF, the directional coupling of some excitons is then shown to be possible with polarization dependence. Away from the NW center (*y* = 0), the excitation scheme can support transverse optical spin angular momentum (t-OSAM) for directional coupling of circularly polarized light, i.e. spin-momentum locking of light with time reversal symmetry^38,39^. The circular polarized light can be emitted from excitons, trions, and biexcitons in the studied structure (Fig. 1b). To investigate the directional coupling, the PL spectra as a function of pump position *y* at both right and left ends are compared and analyzed for directionality (Fig. 4a). The directionality *D* is defined as *D=*(*I*_L_-*I*_R_)/(*I*_L_+*I*_R_), where *I*_L_ and *I*_R_ represent the intensity at the left and right ends, respectively. All these measurements are pumped by a right-circularly polarized *σ*^+^ CW laser at 685 nm with 1 mW power. Primarily, ${{\rm{X}}}_{1}^{-}$ and ${{\rm{X}}}_{2}^{-}$ show clear pump position-dependent directionality: as the position *y* moves from the positive to negative values, the directionality changes its sign. This is due to the t-OSAM with time reversal symmetry as elaborated later. But the directionality for other excitons does not show such features. To take a closer look, the PL spectra at both left and right ends at *y* = 0.4 μm are plotted in Fig. 4b. The directionality of XX^-^ without such features is due to the small valley polarization (Fig. S2); the directionality of XX^0^ is attributed to the extremely low coupling efficiency induced low directionality contrast, and the X^0^ emission is invisible here as discussed above. In contrast, the X_D_ and ${{\rm{X}}}_{{\rm{D}}}^{-}$ with strong emission intensity have smaller directionality without such clear pump-position dependence.

To confirm that the directional coupling of ${{\rm{X}}}_{1}^{-}$ and ${{\rm{X}}}_{2}^{-}$ is resulted from the t-OSAM, the directionality is measured as a function of pump polarization by changing the excitation half-wave plate (see “Methods” section and Fig. S13 for details) as in Fig. 4c. When the polarization changes from linear polarization to right/left circular polarization, the directionality starts from 0 and reaches its positive/negative maximum for both ${{\rm{X}}}_{1}^{-}$ and ${{\rm{X}}}_{2}^{-}$. This observation directly proves the mechanism of the t-OSAM, consistent with other similar t-OSAM configurations^38,39^. In contrast, the X_D_ and ${{\rm{X}}}_{{\rm{D}}}^{-}$ show negligible polarization dependence (see Fig. S14). To corroborate this conclusion, the time-averaged power flows of SPPs toward the NW ends for both in-plane and out-of-plane dipole sources are simulated in Fig. 4d, e. The in-plane dipole is set to right circularly polarized *σ*^+^ for trions and out-of-plane dipole is set to linearly polarized for dark excitons and dark trions (see “Methods” section). For the in-plane *σ*^+^ dipole, the SPP power flows to the right end when the position *y* is set to be positive, and vice versa for the negative y position. For the out-of-plane linear dipole, the SPP power flows evenly for both ends without dependence on the position *y*. This confirms the polarization dependence for X_D_ and ${{\rm{X}}}_{{\rm{D}}}^{-}$. The observed non-zero directionality in Fig. 4a may be explained by the simulation of Fig. S15, where tilted out-of-plane dipole orientation is introduced by the inhomogeneity of the sample. Nevertheless, the reason of inhomogeneity for non-zero directionality needs further experimental investigations. Based on these detailed analyses, the directional coupling via t-OSAM is well established for the trions.

## Discussion

In summary, this work presents versatile optical manipulation of trions, dark excitons, and biexcitons in a monolayer WSe_2_ via contrasting exciton-photon coupling with dependences of dipole orientations, diffusion, and chirality. By leveraging the photonic modes in Ag NWs, the exciton-photon coupling behaves quite differently for various excitons, including ${{\rm{X}}}_{1}^{-}$, ${{\rm{X}}}_{2}^{-}$, X_D_, ${{\rm{X}}}_{{\rm{D}}}^{-}$. and XX^−^ according to the excitonic transition dipole orientations. By the established contrasting exciton-photon coupling, the DOFs of intensity, frequency, and polarization can simultaneously be manipulated on the excitonic spectrum. With the diffusion lengths of various excitons, the exciton-photon coupling could be flexibly tuned to control the full spectral profiles. Via the t-OSAM, the ${{\rm{X}}}_{1}^{-}$, and ${{\rm{X}}}_{2}^{-}$ of in-plane transition dipoles can support directional chiral coupling with polarization dependence. Toward the goal of full manipulation of the comprehensive excitonic spectrum on-demand, this work presents a crucial step for exploiting various excitons with multiple DOFs simultaneously.

For practical applications based on versatile manipulation, the parameters of the photonic structures can be tuned and optimized. In the studied plasmonic NWs here, for example, the diameters of the Ag NW and the thickness of the top hBN can be tuned to adjust the coupling strength and the polarization dependence. As shown in Figs. S5 and S16, the optical contrast for the selective turn-on/turn-off, optical sorting, and directional routing of excitonic emissions could be optimized for realistic optical information process.

## Materials and methods

### Sample fabrication

Monolayer WSe_2_ was prepared by mechanical exfoliation of bulk materials (HQ graphene). The dry transfer method was conducted with a home-build transfer stage. The top hBN thin film (~ 5 nm), WSe_2_, and bottom hBN were picked up in sequence and transferred the heterostructure to a cleaned 285 nm SiO_2_/Si substrate. Chloroform was utilized to dissolve the polycarbonate (PC) film that was used for the 2D materials transfer. The chemically synthesized Ag NWs were first spin-coated on another clean substrate and then transferred to the hBN/WSe_2_/hBN heterostructure by the same dry transfer method. After removing the residual PC film, the sample was deposited with 10 nm aluminum oxide immediately by atomic layer deposition to prevent oxidation of the Ag NW in the air.

### Spectroscopy measurement

For the PL measurement, a CW 685 nm laser was employed to excite the sample. Before reaching the 50× dark-field objective (Olympus, 0.5 NA), the laser passed through a half-wave plate (HWP) and/or a quarter-wave plate (QWP) to alter its polarization. The PL signal from the sample was collected by the same objective and guided to a spectrometer (Andor, Kymera 328i). For the PL spectra in Figs. 1, S2, and S4, the spectrometer was switched to spectrum mode to collect a single spectrum. For the PL spectra in other figures, the spectrometer was switched to image mode. In this mode, the PL signal from the entire Ag NW was diffracted by a grating (300 line/mm) and recorded by an electron multiplying charge-coupled device (Andor, DU970P). The recorded images have two dimensions that contain the information of the *x* position and wavelength, separately. The integration time was 30 s. The spectra from the left and right ends of the Ag NW can be extracted from the same image by selecting different interested areas. All the spectra were collected at a sample temperature of 4 K (Montana, Cryostation S50).

### Electromagnetic simulation

The electromagnetic simulations were carried out using COMSOL Multiphysics 5.2a. Johnson and Christy’s experimental data was adopted to determine the frequency-dependent permittivity of Ag^57^. The refractive index of SiO_2_ was considered as 1.5. In the model, the Ag NW was constructed with a pentagonal cross-section and a corner rounding of 10 nm. The length of the Ag NW is around 8 μm. To simulate an infinite length condition, 200 nm perfect matched layers were added to both ends of the Ag NW. An electric dipole was positioned 5 nm underneath the center of Ag NW to simulate various excitons in WSe_2_. The dipole’s polarization can be controlled by modulating the amplitude and phase between linear dipoles in different directions. Finally, time averaged-power flow SPPs to both ends of the Ag NW were collected to calculate the total coupling strength *ξ* and directionality *D* of different excitons.

### Supplementary information

Supplementary Information
