# Supplementary material for: Versatile optical manipulation of trions, dark excitons and biexcitons through contrasting exciton-photon coupling
Source: Light Sci Appl. 2023 Dec 7;12:295. doi: 10.1038/s41377-023-01338-5 (PMC10700377; doi:10.1038/s41377-023-01338-5)
Supplement: Supplementary file 1 — Supplementary Information [file 41377_2023_1338_MOESM1_ESM.docx]

**Supplementary Information for**

**Versatile optical manipulation of trions, dark excitons and biexcitons through contrasting exciton-photon coupling**

Zhe Li^1#^, Xin-Yuan Zhang^1,2#^, Rundong Ma^1^, Tong Fu^1^, Yan Zeng^1^, Chong Hu^1,2^, Yufeng Cheng^1^, Cheng Wang^1^, Yun Wang^3^, Yuhua Feng^3^, Takashi Taniguchi^4^, Kenji Watanabe^5^, Ti Wang^1*^, Xiaoze Liu^1,2,6*^, Hongxing Xu^1,6,7*^

^1^ School of Physics and Technology, Center for Nanoscience and Nanotechnology, and Key Laboratory of Artificial Micro- and Nanostructures of Ministry of Education, Wuhan University, Wuhan, 430072, China

^2^ Wuhan University Shenzhen Research Institute, Shenzhen, 518057, China

^3^ Institute of Advanced Synthesis, School of Chemistry and Molecular Engineering, Nanjing Tech University, Nanjing 211816, China

^4^ International Center for Materials Nanoarchitectonics, National Institute for Materials Science, 1-1 Namiki, Tsukuba 305-0044, Japan

^5^ Research Center for Functional Materials, National Institute for Materials Science, 1-1 Namiki, Tsukuba 305-0044, Japan

^6^ Wuhan Institute of Quantum Technology, Wuhan 430206, China

^7^ School of Microelectronics, Wuhan University, Wuhan 430072, China

^#^ These authors contributed equally to this work

^*^ Corresponding emails: [wangti@whu.edu.cn](mailto:wangti@whu.edu.cn), [xiaozeliu@whu.edu.cn](mailto:xiaozeliu@whu.edu.cn), [hxxu@whu.edu.cn](mailto:hxxu@whu.edu.cn)

**Contents**

[S1 Sample characterization](#_Toc83975844) 3

S2 [Photoluminescence (PL) spectral analysis and fitting](#_Toc83975836) 4

[S3 Power-law fitting for different exciton species](#_Toc83975836) 5

[S4 Helicity-resolved PL spectra with *σ*^-^ excitation](#_Toc83975836) 6

S5 Simulated coupling strength with various gap distances7

S6 Control sample without hBN 8

[S7 Electric field distribution of the SPP mode](#_Toc83975836) 9

S8 Power-dependent PL spectra from left end of the Ag NW 10

S9 Purcell effect of simulation and TRPL measurement 11

S10 Position-dependent PL spectra from the left end 12

S11 Details for the extraction of diffusion lengths13

S12 Excluding the effect of SiO_2_/Si substrate waveguide case 15

S13 Schematics of experimental setup17

S14 Polarization dependent directionality of $\text{X}_{\text{D}}$ and $\text{X}_{\text{D}}^{\text{-}}$ 18

S15 Simulation for linear dipoles in *xz* plane19

S16 Simulated coupling strength varying the diameters of the NW 20

References 21

**S1 Sample characterization**


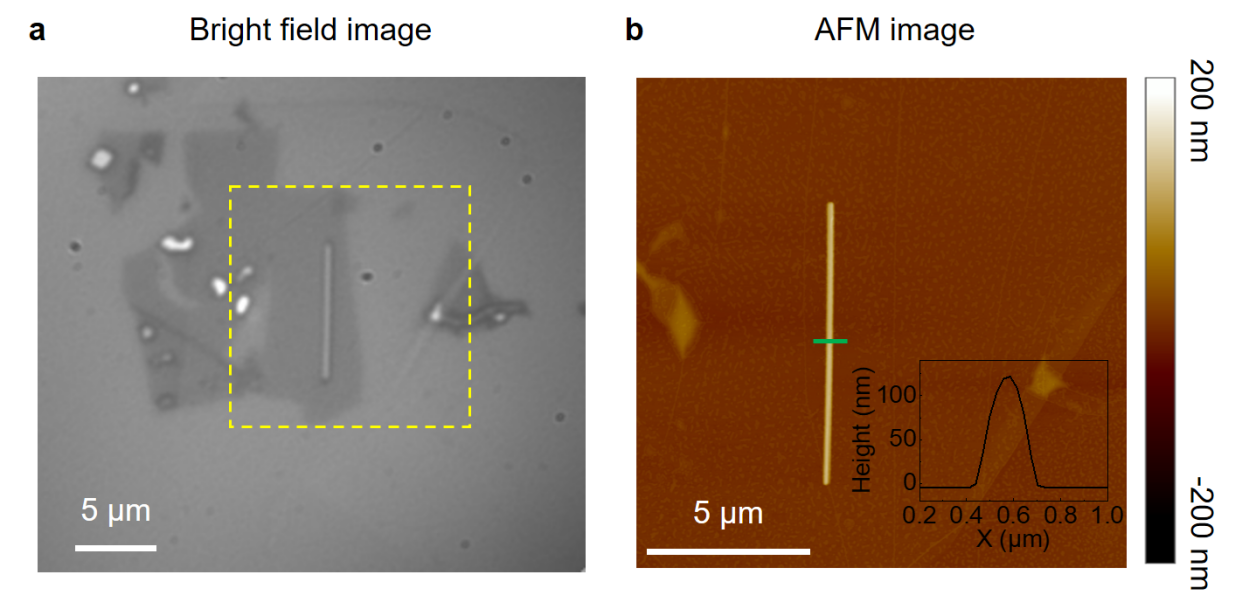


**Fig. S1** **a,** Bright filed image of the Ag NW-WSe_2_ hybrid structure. **b,** Atomic force microscopy (AFM) image of the sample. The AFM scanning area is marked as the yellow dashed square in **a**. The height of Ag NW along the green line is shown in the inset. The diameter of Ag NW is determined to be ~140 nm from the AFM.

**S2 Photoluminescence (PL) spectral analysis and fitting**


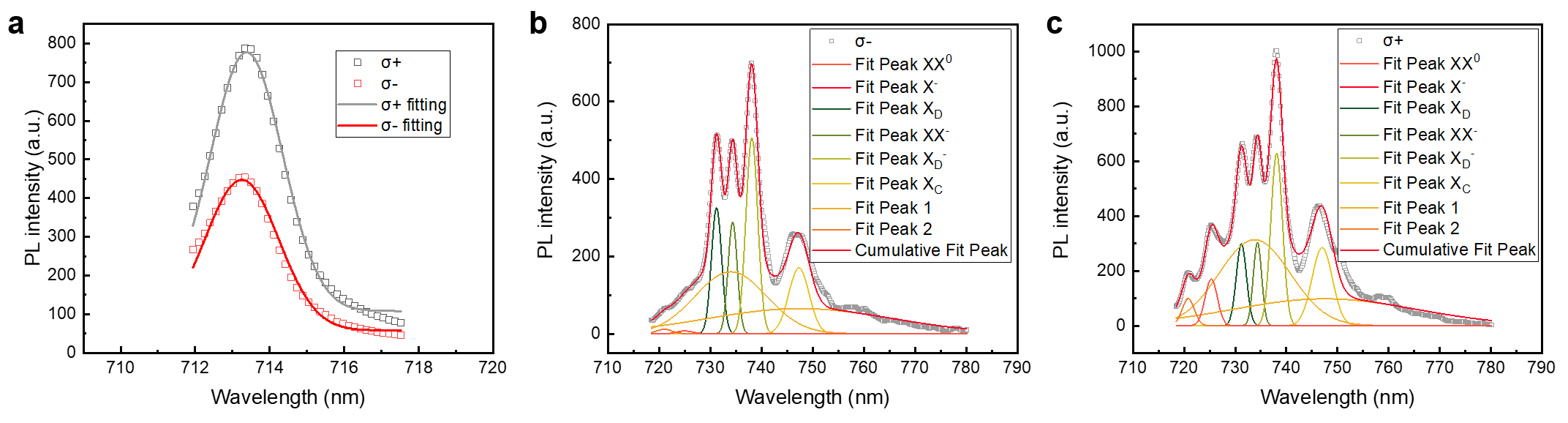


**Fig. S2** **a,** Helicity-resolved PL spectra of the neutral exciton ($\text{X}\text{0}$) by *σ*^+^ pump at 685nm CW laser in **Fig. 1c**. The fitted results for *σ*^+^ and *σ*^-^ detections are shown as gray and red lines. **b** and **c** are the fitted results of other exciton species of *σ*^-^ and *σ*^+^ detection, respectively.

|  | $\text{X}^{0}$ | $\text{XX}^{0}$ | $\text{X}^{\text{-}}$ | $\text{X}_{\text{D}}$ | $\text{X}\text{X}^{\text{-}}$ | $\text{X}_{\text{D}}^{\text{-}}$ |
| --- | --- | --- | --- | --- | --- | --- |
| $\rho$ | 27% | 79% | 92% | -4% | 3% | 11% |

**Table S2** The degree of circular polarization for different exciton species calculated from **Fig. S2**. The degree of circular polarization of $\text{X}^{\text{-}}$ is the highest, with a value of 92%**.** X_D_, X$\text{X}^{\text{-}}$ and $\text{X}_{\text{D}}^{\text{-}}$ shows negligible values of *ρ*. The degree of polarization and peak position for these excitons are consistent with previous reports ^1-4^.

We note that defects are inevitably introduced during the sample preparation process, so that defect-related emissions make it more difficult to obtain the actual degree of circular polarization of specific excitons. To obtain a more accurate degree of circular polarization, multi-peak fitting is needed to excluded the influence of background (broad peak 1 and 2). The techniques have also been reported in previous studies such as Ref. 53 in the main text, and are now adopted as a quite necessary but reasonable procedure to look into the spectral degree of circular polarization.

**S3 Power-law fitting for different exciton species**


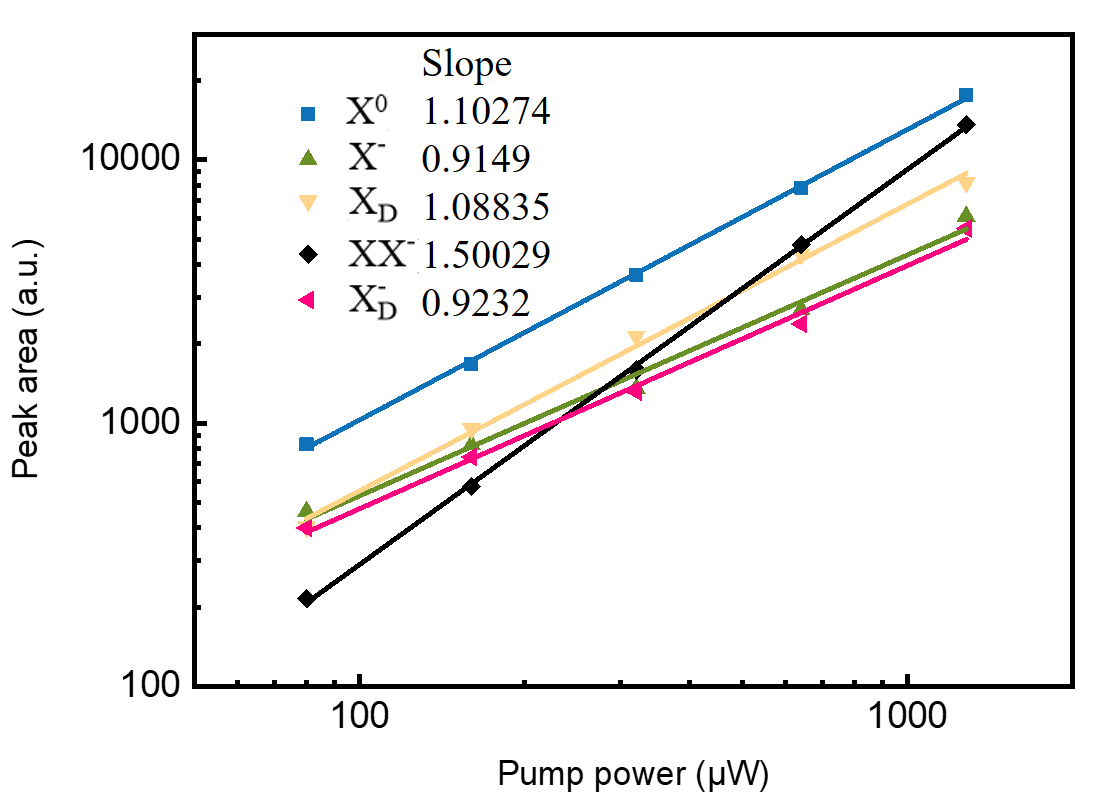


**Fig. S3** Power-law fitting for different exciton species of WSe_2_ without Ag NWs. The power-law fitting gives exponents of 1.10, 0.91, 1.09, 0.92 for $\text{X}\text{0}$, $\text{X}^{\text{-}}$, $\text{X}_{\text{D}}$ and $\text{X}_{\text{D}}^{\text{-}}$, respectively. The exponents of $\text{X}\text{X}^{\text{-}}$ deviated from 2 was attributed to the partial equilibrium between the biexciton and the constituent exciton. The power-law results for different exciton species are consistent with previous reports ^1-4^.

**S4 Helicity-resolved PL spectra with *σ*^-^ excitation**


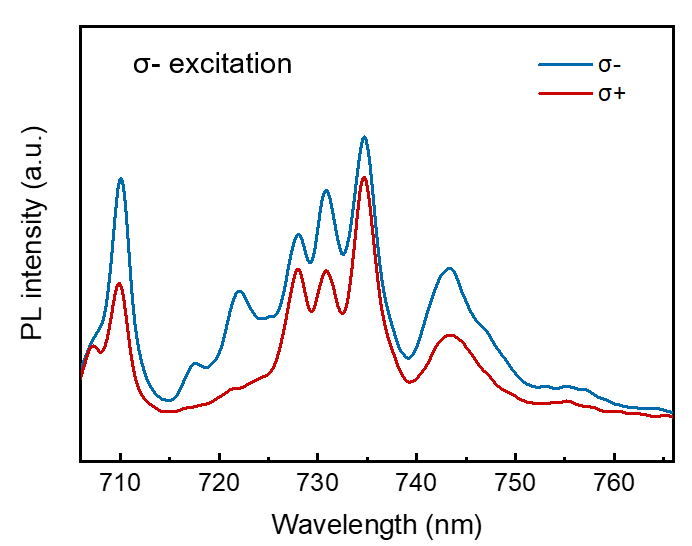


**Fig. S4** PL spectra of monolayer WSe_2_ far away from Ag NW at 4 K**.** The blue and red lines are the PL signal with *σ*^-^ and *σ*^+^ detection when excited by a *σ*^-^ polarized laser of 685nm. The helicity-resolved PL spectra are consistent with the results shown in **Fig. 1b**.

**S5 Simulated coupling strength with various gap distances**


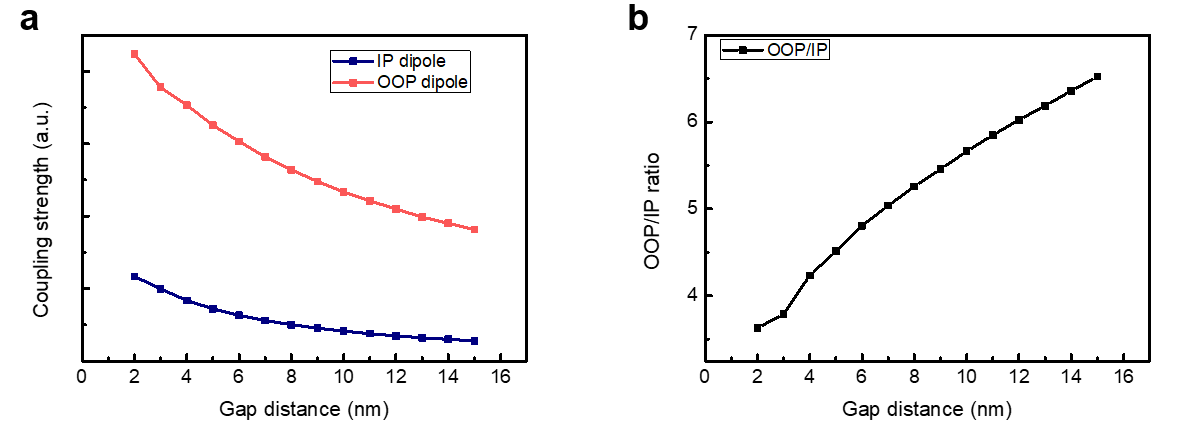


**Fig. S5** The simulation of coupling strength between dipoles and Ag NW with various gaps. **a**, the simulated coupling strength of in-plane (IP) and out-of-plane (OOP) dipoles at the edge of a 110 nm diameter Ag NW as a function of gap distance. The coupling strength for both OOP and IP dipoles decays with distance, and the strength for OOP keeps higher than that of IP. **b** is the ratio of coupling strength between OOP and IP dipoles with increase of gap distance. The ratio of the two is positively correlated with the gap distance. The ratio between the two can be effectively modulated by adjusting the value of the gap distance.

**S6 Control sample without hBN**


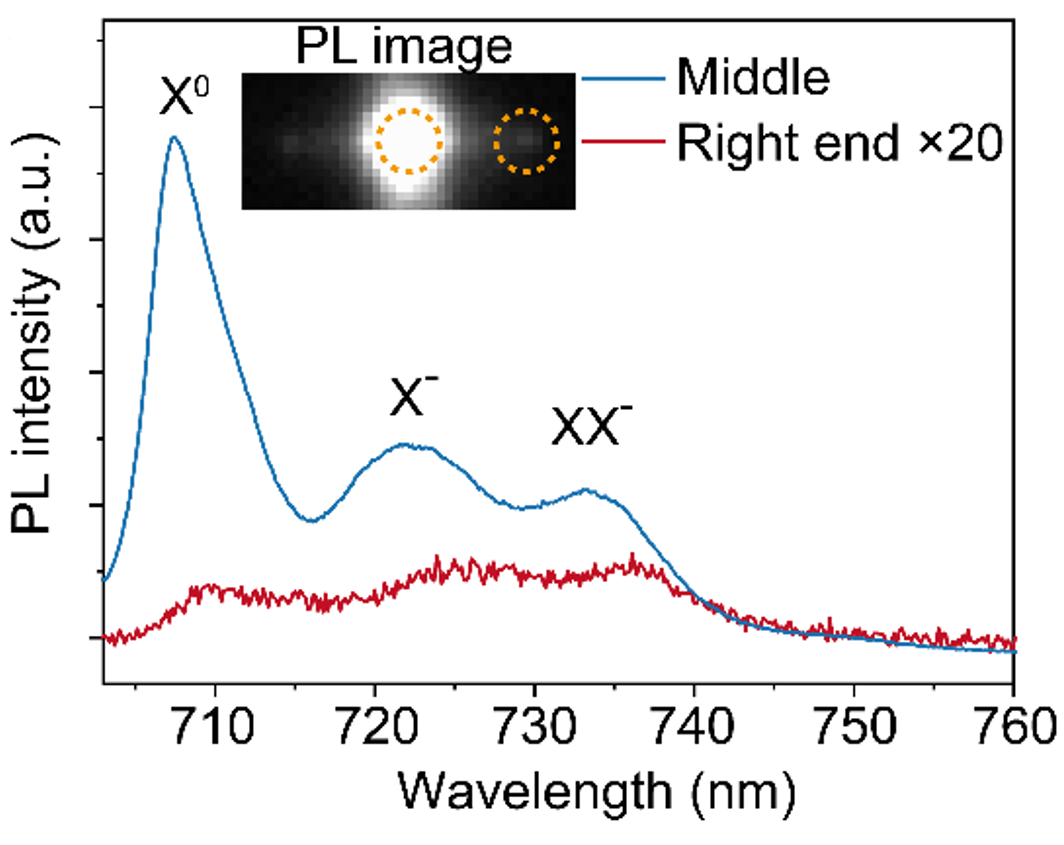


**Fig. S6** In-situ (collected at the middle point of NW) and propagated PL spectra (collected at right end) for a sample without hBN, the insets show the corresponding PL image.

**S7** **Electric field distribution of the SPP mode**


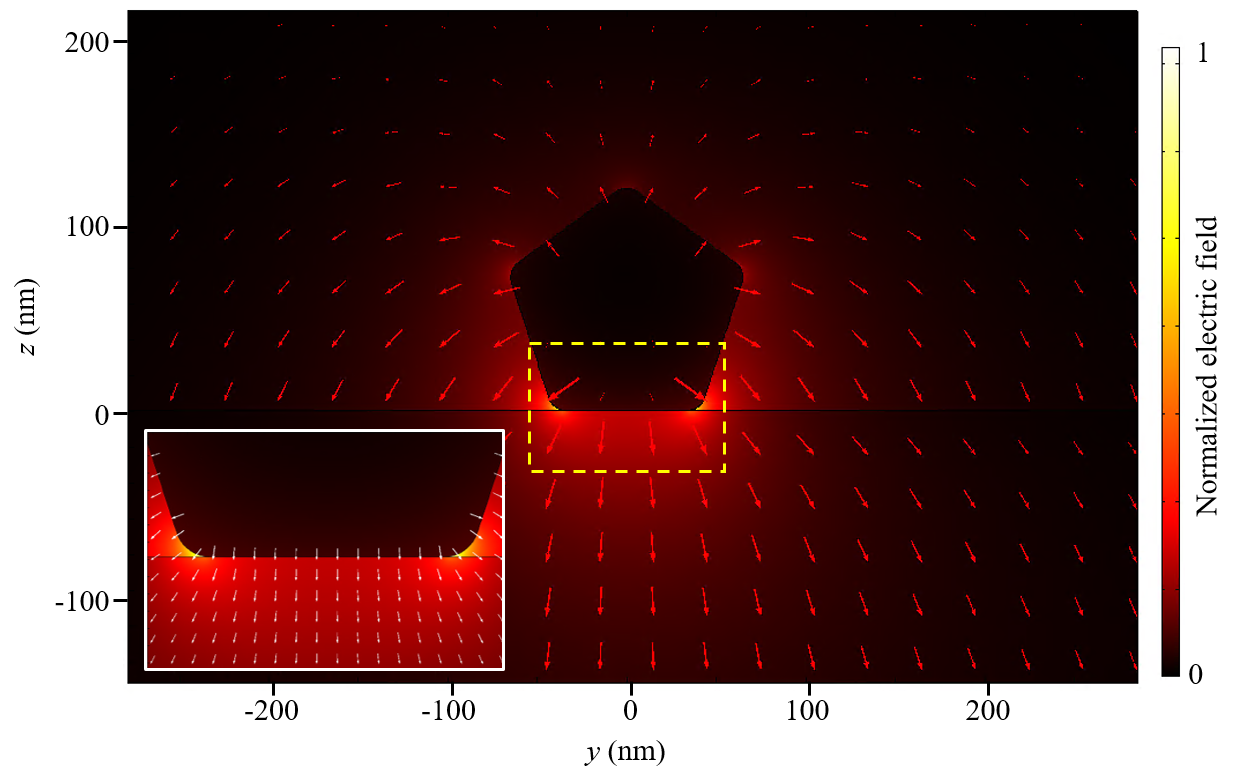
**Fig. S7** Electric field distribution of the propagating mode for Ag NW with a diameter of 140 nm. The inset is an enlarged view of the yellow dashed square area. The *z* component of the electric field underneath the Ag NW is much stronger than the *y* component, resulting in a larger coupling strength with an out-of-plane dipole. Here the arrows represent the electric field directions.

**S8 Power-dependent PL spectra from left end of the Ag NW**


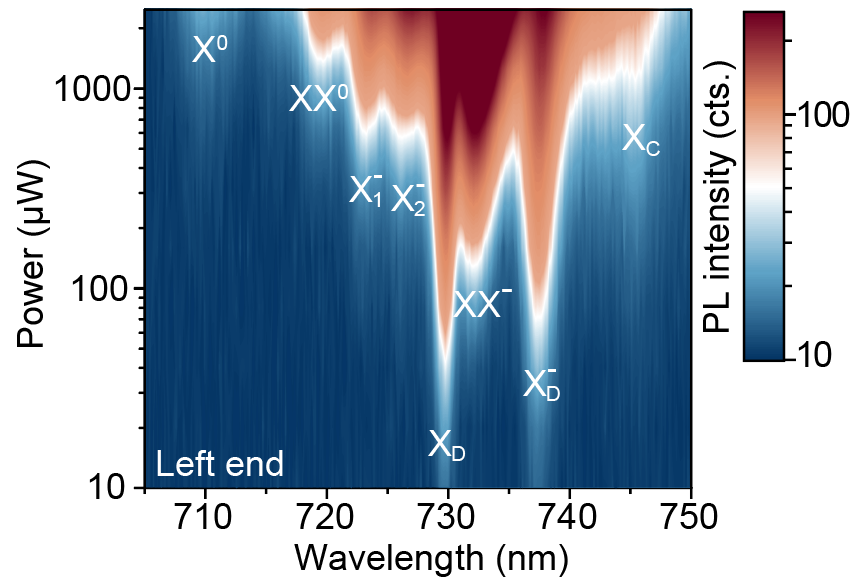


**Fig. S8** The PL spectra from left end of the Ag NW. The data were obtained with the same excitation of **Fig. 2b** and **2d** in the main text. The PL spectrum from left end also reveals the differences from the in-situ spectra, such as the narrower excitonic linewidths and stronger dark exciton intensity.

**S9 Purcell effect of simulation and TRPL measurement**


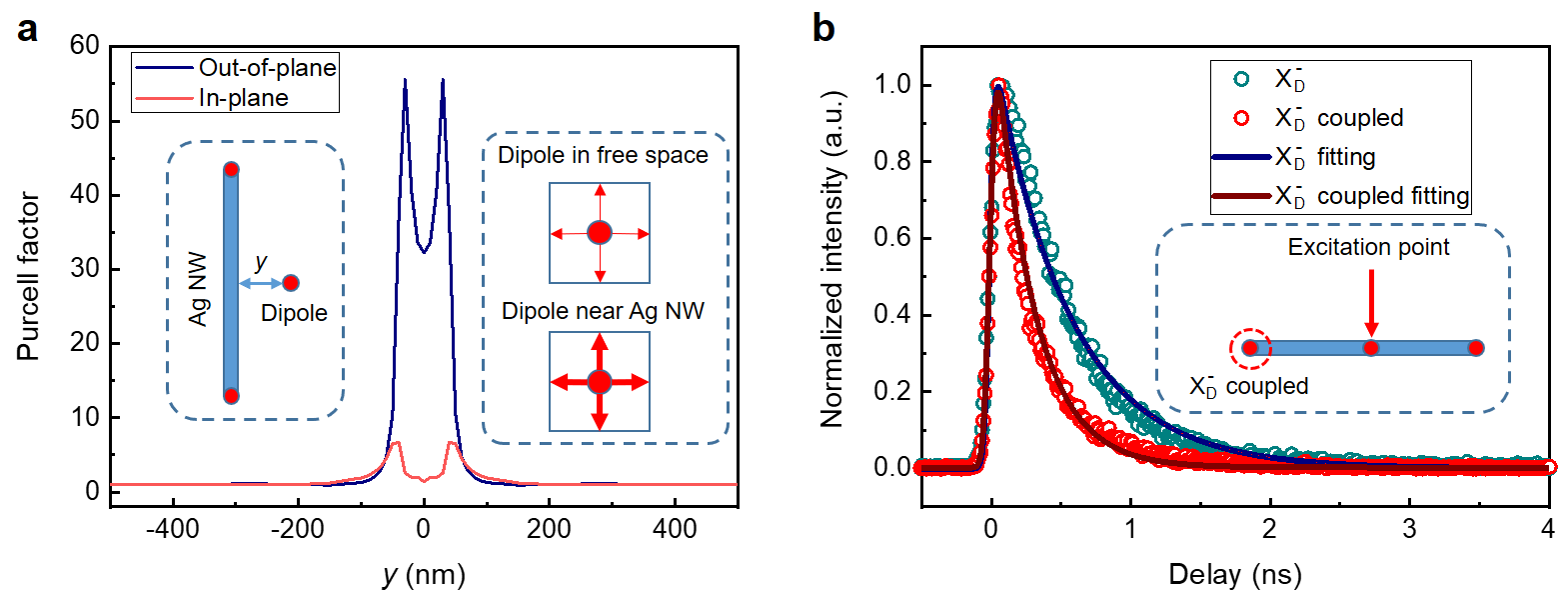


**Fig. S9** **a**, Simulated Purcell factor of out-of-plane (blue) and in plane dipole (pink). The right inset shows the schematics of the model where the density of states (DOS) in each case is integrated, the left inset shows the schematics of the *y* position with respect to the NW center. **b**, TRPL spectra of uncoupled (off the NW) and coupled $\text{X}_{\text{D}}^{\text{-}}$ (on the NW) of WSe_2_. The lifetimes for uncoupled and coupled $\text{X}_{\text{D}}^{\text{-}}$ are ~ 548 ps and ~ 280 ps, respectively, indicating a spatially averaged Purcell factor of 1.96. Note the experimental configuration of the “coupled dark exciton” as the inset refers to collecting coupled signals at NW ends and exciting at NW center.

Simulation and experimental details:

The simulation of Purcell factor was carried out using COMSOL Multiphysics 5.2a. As illustrated by the inset of Fig. S9a, a small cage is added in the model to integrate the energy outflow from the electric dipole. The electric dipole was positioned 5 nm underneath the center of Ag NW. The position of the dipole was scanned from -500 nm to 500 nm relative to the Ag NW in the *y* direction. Finally, the integrated energy outflow from each point was divided by the integrated energy outflow of no photonic coupling case to extract the Purcell factor.

A picosecond pulsed laser with an excitation of 400 nm and a repetition rate of 40 MHz was used to excite the sample for time-resolved photoluminescence (TRPL) measurements, which was focused by a 50× (NA ~0.5) objective. The PL emission was collected with the same objective, dispersed with a monochromator (Andor Technology) and detected by a time-correlated single photon counting (TCSPC) system. The pump power was 25 μW. TRPL traces are fitted with instrumental response function convoluted with a single exponential decay:

$$\begin{aligned} I_{t}=A_{1}e^{-\frac{t}{\tau_{1}}} \text{(}\text{S1}\text{)} \end{aligned}$$

PL intensity $I_{t}$ is a function of delay time *t*, *τ*_1_ is the time constant for the exponential decay, *A*_1_ is amplitude.

**S10 Position-dependent PL spectra from the left end**


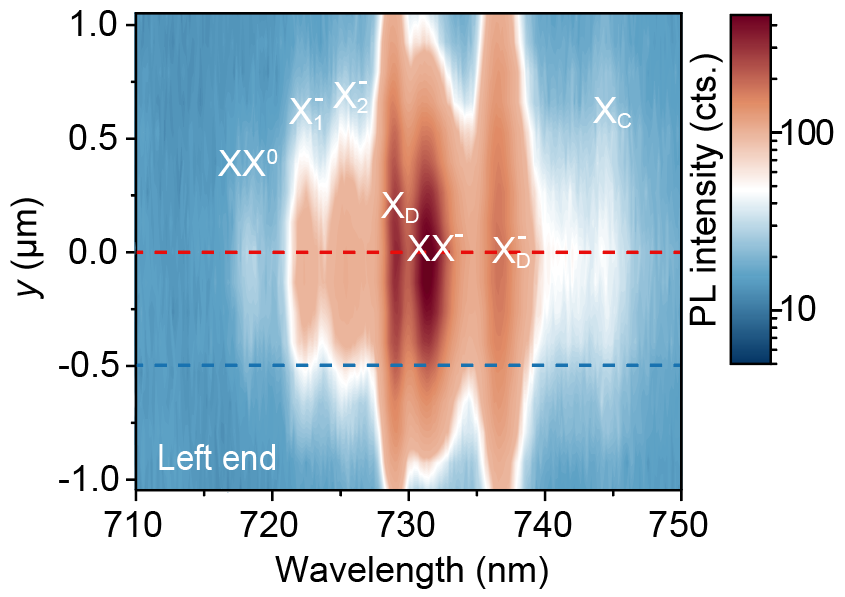


**Fig. S10** Color map of PL spectra as a function of the distance *y* from the left end. The variable *y* represents the distance from the laser spot to the Ag NW. As the absolute value of *y* increases, the PL intensity decreases.

**S11 Details for the extraction of diffusion lengths**

As discussed in the main text, the steady state exciton distribution can be described by the following formula:

$$\begin{aligned} \text{n}\text{(}\text{r}\text{) }\propto\text{ }\int_{\text{-∞}}^{\text{∞}} \text{ }\text{K}_{\text{0}}\text{(}\text{r}^{\text{'}}\text{/}\text{L}_{\text{X}}\text{)}\text{e}^{{\text{-(}\text{r}\text{ }\text{-}{\text{ }\text{r}}^{\text{'}}\text{)}}^{\text{2}}\text{/}\text{w}^{\text{2}}}\text{d}\text{r}^{\text{'}} \text{(S2)} \end{aligned}$$

where $\text{L}_{\text{X}}\text{ }\text{= }\sqrt{\text{D}_{\text{X}}\text{τ}_{\text{X}}}$ is the diffusion length. While the diffusion constants $\text{D}_{\text{X}}$ for these exciton species are similar (around 1 ~ 10 cm^2^/s) ^5,6^, the lifetimes $\text{τ}_{\text{X}}$ differ a lot ^7,8^. Due to the spin-forbidden transition, the $\text{τ}_{\text{X}}$ for $\text{X}_{\text{D}}$ and $\text{X}_{\text{D}}^{\text{-}}$ are more than ten times longer than the bright excitons, leading to much longer diffusion lengths.

Based on previous reports, we estimate the $\text{L}_{\text{X}}$ for $\text{X}_{\text{D}}$ and $\text{X}_{\text{D}}^{\text{-}}$ to be a few hundred nanometers and the $\text{L}_{\text{X}}$ for $\text{X}_{\text{1}}^{\text{-}}$, $\text{X}_{\text{2}}^{\text{-}}$ and $\text{X}\text{X}^{\text{-}}$ to be tens of nanometers. The $\text{L}_{\text{X}}$ for $\text{X}_{\text{1}}^{\text{-}}$, $\text{X}_{\text{2}}^{\text{-}}$ and $\text{X}\text{X}^{\text{-}}$ are too small to be quantified considering the relatively big beam spot (diameter ~ 1 μm). Meanwhile, as $\text{X}\text{X}^{\text{-}}$ has a higher power-law exponent (Fig. S3), its initial exciton distribution is more concentrated in the beam spot center area, which leads to a narrower distribution.

To extract the $\text{L}_{\text{X}}$ for $\text{X}_{\text{D}}$ and $\text{X}_{\text{D}}^{\text{-}}$, we use the exciton distribution of $\text{X}_{\text{1}}^{\text{-}}$ as the initial excitation condition as $\text{X}_{\text{1}}^{\text{-}}$ have very short $\text{L}_{\text{X}}$ and similar power-law exponents with the two dark excitons (Fig. S3). By fitting the distribution of $\text{X}_{\text{1}}^{\text{-}}$ with the Gaussian function, we get a $\text{w}$ value of 0.89. Then Eq. (S1) is employed to obtain the only remaining variable $\text{L}_{\text{X}}$ for $\text{X}_{\text{D}}$ and $\text{X}_{\text{D}}^{\text{-}}$. The extracted $\text{L}_{\text{X}}$ for $\text{X}_{\text{D}}$ and $\text{X}_{\text{D}}^{\text{-}}$ are 0.59 ± 0.10 μm and 0.84 ± 0.13 μm, respectively.


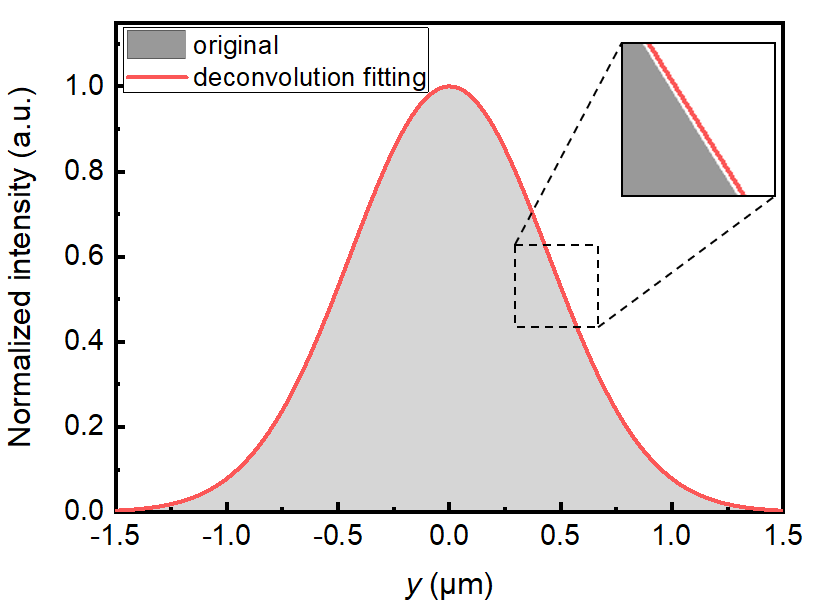


**Fig. S11** The original and deconvolution fitted diffusion profiles. The deconvolution includes the parameter of 140 nm diameter of the Ag NW.

Furthermore, the width of the Ag NW may have an effect on the $\text{L}_{\text{X}}$ extraction. We use 140 nm as the width to deconvolution fit the data. The deconvolution fitting casts negligible effect on the result. As shown in Fig. S11, this deconvolution fitting is almost the same as the original fitting without deconvolution. The diffusion profile of the deconvolution fitting shows the full width at half maximum (FWHM) is increased to 1.044 μm which is only 4 nm wider than the originally fitted profile. The output diffusion length for $\text{X}_{\text{D}}^{\text{-}}$ is still 0.84 μm with negligible difference.

**S12 Excluding the effect of SiO_2_/Si substrate waveguide case**


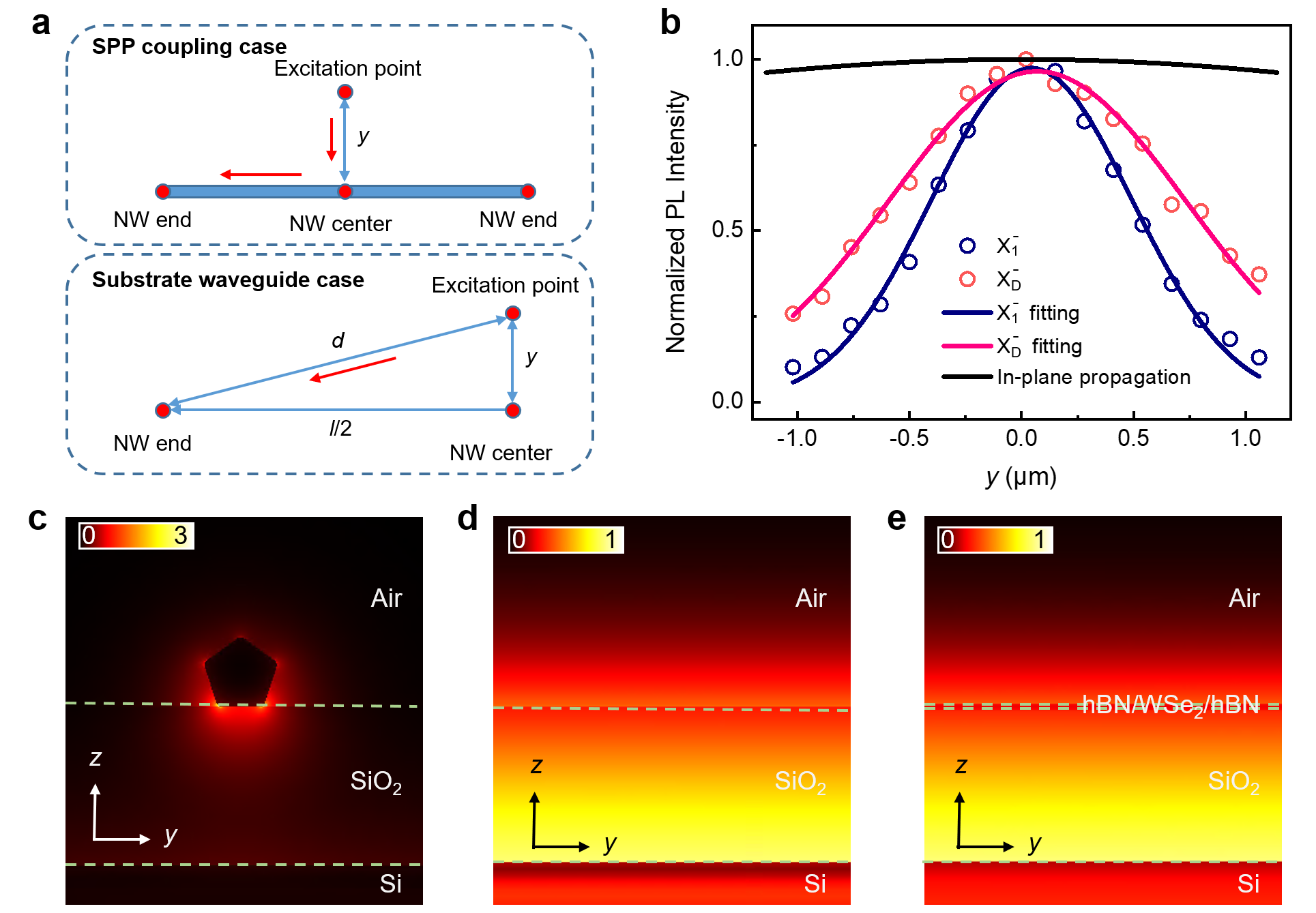


**Fig. S12** **a**, Schematics of the two cases of SPP coupling and substrate waveguide. **b**, Data fitting with the models of these two cases. Electric field distributions for the modes in **c**. the SPP coupling case and the substrate waveguide case without (**d**) and with the heterostructure (**e**).

The in-plane propagation would happen when the underneath substrate is taken as the waveguide for the excitonic emissions of monolayer WSe_2_. We sketch two cases in the *y*-coordinated schematic of Fig. S12a: the case of SPP coupling process through the Ag NW (SPP coupling case), and the one of in-plane propagation via substrate waveguide (substrate waveguide case). In the SPP coupling case, various exciton species firstly diffuse and couple with the Ag NW, and then propagate along the NW to finally scatter out at NW ends. The PL intensity thus mainly depends on the *y* coordinate via an exciton diffusion model with parameters of diffusion lengths (Equations (1) and (2) in the main text). In the substrate waveguide case, the in-plane propagation is isotropic and thus the scattered-out emission mainly depends on the distance *d* between the excitation point and the end of the NW without any nonlinear processes. In this case, the emission intensity follows:

$$\begin{aligned} I\propto\frac{1}{d}=\frac{1}{\sqrt{{\frac{l}{4}}^{2}+y^{2}}} \text{(}\text{S3}\text{)} \end{aligned}$$

where *l* is the length of the Ag NW. These cases are modeled to fit the experimental data (Fig. S12b). Apparently, the SPP coupling model can fit well with the data and the extracted exciton diffusion lengths. In contrast, the waveguide case cannot fit the data, and is hence excluded.

These two cases of coupling can also be simulated by COMSOL, whose mode analysis is shown in Fig. S12. In the SPP coupling case (Fig. S12c), the electric field is tightly bound around the Ag NW. In the substrate waveguide case (Fig. S12d-e), the electric field for the fundamental waveguide mode is isotropic and uniform in any direction. These field profiles unambiguously point out the SPP coupling case is much more efficient, and much more directional along the NW, as observed in the experiments. Moreover, the effective refractive index *n*_eff_ of these two cases is simulated to be 1.5141-0.0540i (Fig. S12c) and 1.4449-0.0016i (Fig. S12d), respectively. Compared with the SPP case, the substrate waveguide case shows a tiny imaginary part of *n*_eff_ for negligible propagation loss, which is also not the observed scenario in this work. Besides, the van der Waals heterostructure used in the experiment can only slightly modified the mode (Fig. S12e) and will not affect this conclusion.

Based on the above experimental and simulation analysis, the possibility is totally excluded that the experimental phenomenon may come from the in-plane propagation via substrate waveguide.

**S13 Schematics of experimental setup**


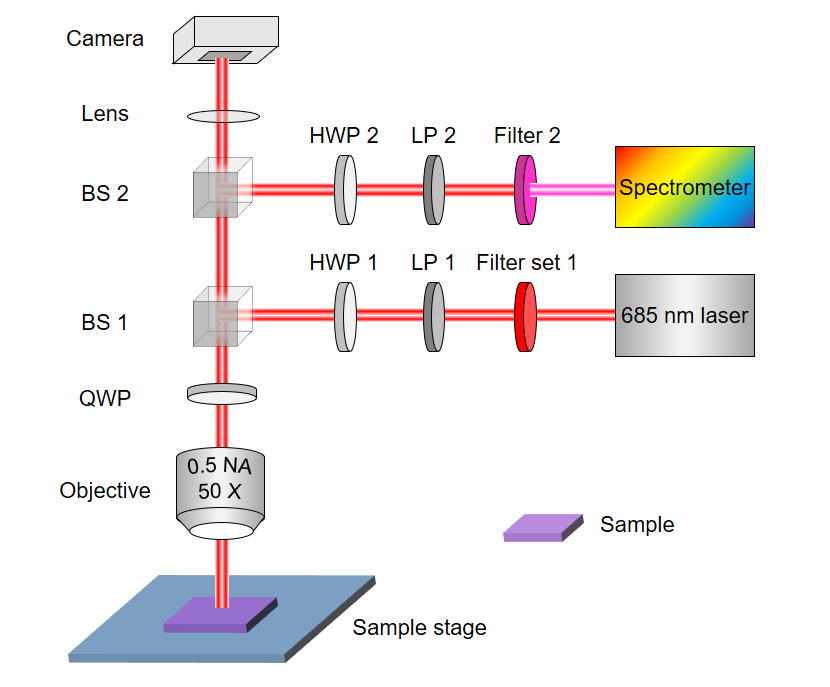


**Fig. S13** Schematics the optical setup for spectral measurement. Filter set 1 contains two filters: 700 nm short pass and 690 $\pm$ 5 nm band pass. Filter 2: 700 nm long pass filter. LP (linear polarizer), HWP (half-wave plate) and QWP (quarter-wave plate) are used to alter the polarization of the excitation and collection. BS: non-polarized beam splitter.

**S14 Polarization dependent directionality of** $\text{X}_{\text{D}}$ **and** $\text{X}_{\text{D}}^{\text{-}}$


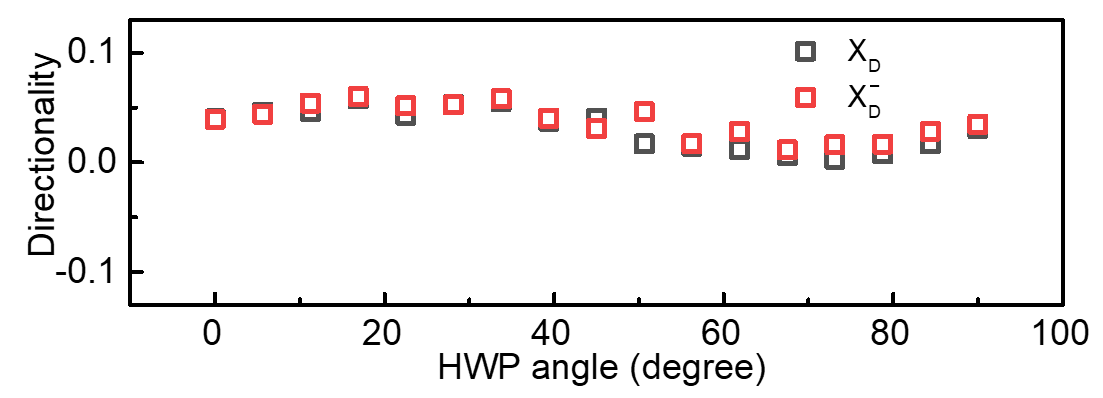


**Fig. S14** Polarization dependent directionality of $\text{X}_{\text{D}}$ and $\text{X}_{\text{D}}^{\text{-}}$, the polarization of the laser is modified by a half wave-plate (HWP). Compared with $\text{X}_{\text{1}}^{\text{-}}$ and $\text{X}_{\text{2}}^{\text{-}}$, the two dark excitons show negligible polarization dependence. The slight polarization dependence may come from the background change from other excitons.

**S15 Simulation for linear dipoles in *xz* plane**


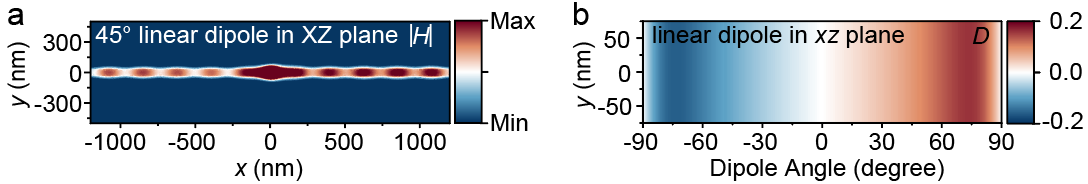


**Fig. S15** COMSOL Multiphysics modeling of the directionality for dipoles in *xz* plane. **a**, Magnetic field distribution of SPPs excited by the 45$^{\circ}$tilted linear dipole. **b** is the simulated dipole angle dependent directionality (*D*) for linear dipole in *xz* plane. The directionality keeps increase as the angle increase and reaches its maximum at around 70$^{\circ}$.

**S16 Simulated coupling strength varying the diameters of the NW**


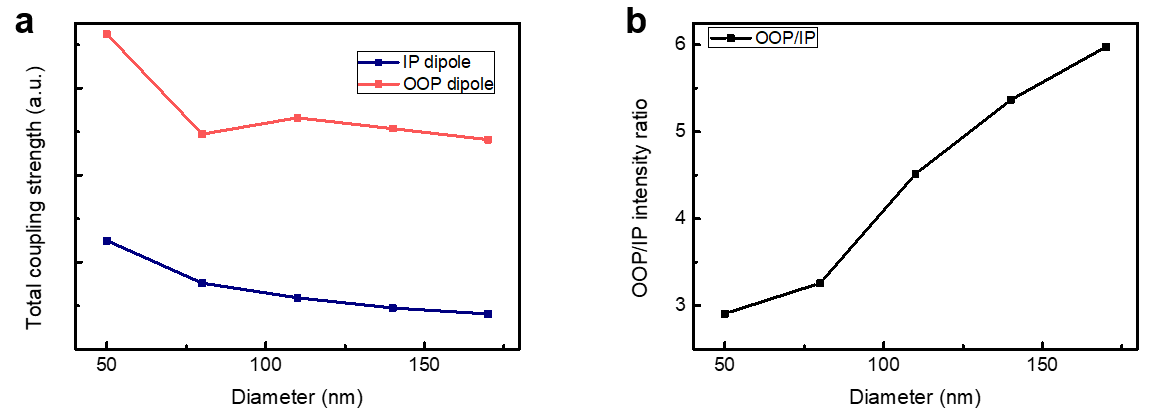


**Fig. S16** The simulated coupling strength between dipoles and Ag NW with various diameters of NWs. **a**, the simulated coupling strength between OOP(IP) dipole and Ag NWs with different diameters. The coupling strength of the OOP (IP) dipoles decreases with increasing diameter of the NW. **b** is the coupling strength ratio of OOP and IP with various diameters. The ratio of the two is positively correlated with the diameter, which provides another tool to modulate the ratio between them.

**References**

1 Li, Z. P. *et al.* Revealing the biexciton and trion-exciton complexes in BN encapsulated WSe_2_. *Nature Communications* **9**, 3719 (2018).

2 Liu, E. F. *et al.* Landau-quantized excitonic absorption and luminescence in a monolayer valley semiconductor. *Physical Review Letters* **124**, 097401 (2020).

3 Steinhoff, A. *et al.* Biexciton fine structure in monolayer transition metal dichalcogenides. *Nature Physics* **14**, 1199-1204 (2018).

4 Ye, Z. L. *et al.* Efficient generation of neutral and charged biexcitons in encapsulated WSe_2_ monolayers. *Nature Communications* **9**, 3718 (2018).

5 Wagner, K. *et al.* Nonclassical exciton diffusion in monolayer WSe_2_. *Physical Review Letters* **127**, 076801 (2021).

6 Zipfel, J. *et al.* Exciton diffusion in monolayer semiconductors with suppressed disorder. *Physical Review B* **101**, 115430 (2020).

7 Robert, C. *et al.* Fine structure and lifetime of dark excitons in transition metal dichalcogenide monolayers. *Physical Review B* **96**, 155423 (2017).

8 Zhang, X. X. *et al.* Magnetic brightening and control of dark excitons in monolayer WSe_2_. *Nature Nanotechnology* **12**, 883-888 (2017).
